# Novel cryopreservation medium for enhanced stability of T cells at −80°C

**DOI:** 10.3389/frhem.2024.1346627

**Published:** 2024-06-17

**Authors:** WenXuan Zhou, Chen Wang, Yao Shi, Yi Pan, XiaDuo Meng, XunLei Kang, Xu Han

**Affiliations:** 1 Center for Precision Medicine, Department of Medicine, University of Missouri School of Medicine, Columbia, MO, United States; 2 Division of Hematology and Oncology, Department of Medicine, University of Missouri School of Medicine, Columbia, MO, United States; 3 Ellis Fischel Cancer Center at The University of Missouri (MU) Health Care, University of Missouri, Columbia, MO, United States; 4 Wake Forest Institute of Regenerative Medicine, School of Medicine, Wake Forest University, Winston-Salem, NC, United States; 5 Mammalian Cryopreservation Division, CryoCrate LLC, Columbia, MO, United States

**Keywords:** cryopreservation, immunotherapy, T cell, DMSO, C80EZ

## Abstract

The increasing demand for immune cell applications, both in clinical settings and in research laboratories, has highlighted the critical need for cryopreservation (banking) methods for T cells. While conventional techniques such as freezing with liquid nitrogen remain prevalent, they pose significant challenges including high equipment costs, safety considerations, and logistical hurdles in transportation. Our cryopreservation medium, C80EZ^®^, represents a novel approach, leveraging biocompatible polysaccharides as cryoprotectants to enable safe storage at −80°C. This paper presents a comprehensive series of tests assessing the effectiveness of C80EZ^®^ in shielding T cells from the detrimental effects of cryopreservation. Importantly, our findings demonstrate that C80EZ^®^ not only ensures the survival of T cells, with a particular emphasis on preserving the CD8^+^ subsets, but also maintains their critical function in targeting and eliminating cancer cells.

## Introduction

T cells, which are crucial in cancer therapy, activate immune functions upon pathogen invasion (1–3), and the immune function will be exercised by the release of tumor necrosis factor alpha (TNF-α), interferon gamma (INF-γ), interleukin 2 (IL-2), and other cytokines (4, 5). The surge in the use of immune cells, such as the production of chimeric antigen receptor (CAR) T cells (6, 7), has underscored the importance of cryopreservation, a challenge that still plagues T-cell immunotherapy (8–12). Cryopreserved immune cells find widespread use in adoptive transfer, prompting a need for efficient cryopreservation methods (13, 14). Despite notable successes in multiple practical applications, cryopreservation still introduces challenges such as impaired cytotoxicity, altered gene expression, and changes in the T-cell phenotype (15–17). It remains crucial to enhance the efficacy of cryopreservation for clinical purposes (6, 18–20).

Moreover, the rising demand for allogeneic T-cell clinical transplantation has intensified the need for improved storage technologies (21) in order to reduce the costs of equipment and labor and to mitigate risks during transportation (22, 23). Presently, due to the thermodynamic instability of conventional cryoprotective solutions that solely rely on the use of small-molecule cryoprotectants such as dimethyl sulfoxide (DMSO) at −80°C, clinical studies predominantly rely on storage in cryogenic liquid nitrogen (LN2) facilities that operate at a temperature range between −196°C (in the liquid phase) and approximately −120°C (in the gas phase). However, the use of LN2 presents logistical and operational challenges. Addressing the core issue of the stability of the medium at −80°C becomes pivotal for effective cryopreservation (24, 25). Considering the vulnerability of T cells to the damaging effects involved in cryopreservation procedures, maintaining both cell vitality and functionality after long-term storage at −80°C is technically challenging for conventional cryopreservation media. This difficulty primarily arises because conventional cryopreservation solutions—which typically contain small-molecule cell-permeating cryoprotectants such as DMSO and ethylene glycol, as well as macromolecular cryoprotectants such as fetal bovine serum (FBS) components and various linear or branched synthetic polymers such as polyethylene glycol and dextran—undergo ice recrystallization (26–28). This process spontaneously occurs at temperatures higher than those in LN2 storage, which leads to the gradual formation of enlarged ice crystals. These crystals, growing from smaller ones formed during the initial freezing, inflict severe mechanical damage on regular mammalian cell types. Numerous mammalian cell types cryopreserved in conventional media have shown considerable viability loss after short-term storage at −80°C, with complete viability loss over longer periods (e.g., 1 year) (29–32).

No systematic research has been conducted to comprehensively evaluate the impact of storage at −80°C on the post-thaw viability, the proliferation efficiency, and, more importantly, the cytotoxicity of primary T cells. In this work, we aimed to not only address these research gaps but also demonstrate a promising solution to provide a new direction in immune cell cryopreservation and transportation that can overcome the technical challenges in supply chain management for immunotherapies based on the use of T cells and other immune cells. In a word, this study introduces a novel polymer-based cryopreservation medium uniquely designed to enhance the outcome of the cryo-storage of T cells at −80°C by preventing the recrystallization process and its associated damaging effects.

## Materials and methods

### Cells and media

T cells were isolated from human peripheral blood (obtained from the American Red Cross) using the MojoSortTM Human T-Cell Isolation Kit (BioLegend, San Diego, CA, USA). Following successful isolation, the cell count and viability were determined. Viable T cells were cryopreserved at −80°C using standard cryopreservation medium (control: 95% FBS + 5% DMSO] and C80EZ^®^ (CryoCrate LLC, Columbia, MO, USA) containing 5% DMSO. The cells were suspended in the cryopreservation medium and placed in a Corning CoolCell LX Cell Freezing Container (Sigma-Aldrich, St. Louis, MO, USA). Subsequently, the freezing container was transferred to a −80°C freezer (Fermalogic, Chicago, IL, USA).

### Cryopreservation medium test and thawing procedures

To assess the efficacy of the cryopreservation medium, the T cells were taken out of the freezer and subjected to a 37°C water bath for 3 min after being cryopreserved at −80°C for 2 months. A gentle swinging motion was applied to the tubes containing cells in the water bath and the water level kept under the cap. Once the ice in the tubes became imperceptible, the solution containing cells was transferred into a 10-mL complete culture medium drop by drop to facilitate the recovery of cells from freezing (21, 33). Of the culture medium, 1 mL was used to wash the tube and was combined with the previous medium to make sure all cells in the cryotube could be harvested. Subsequently, the thawed cells underwent centrifugation at 150 × g for 10 min, following which the supernatant was removed and the pellet resuspended in standard growth medium. These cells were then prepared for testing.

### Viability and proliferation test

To evaluate the viability of T cells post-thawing from −80°C, the number of freshly thawed cells was calculated. An appropriate number of cells was resuspended and then stained with 7-aminoactinomycin D (7AAD) on ice for 3 min. The cells were then centrifuged and resuspended with a staining buffer. Subsequently, the vials containing the stained cells were loaded into a flow cytometer (BD Accuri^™^ C6 Plus) to calculate the proportion of live cells. The data were collected and analyzed with FlowJo software.

To evaluate the proliferation of T cells post-cryopreservation from −80°C, we cultured the T cells in ImmunoCult^™^-XF T Cell Expansion Medium (STEMCELL Technologies, Vancouver, Canada) for 6 days. The cell numbers were calculated at 48, 96, and 144 h after seeding in plates. Prior to culturing, the T cells were stimulated using CD3/CD28 beads (Gibco by Thermo Fisher Scientific, Waltham, MA, USA) overnight, and human interleukin 2 (IL-2) cytokine was supplied during the culture progression. The proliferation of T cells was presented by the expansion fold: Expansion Fold = Harvest cell number/Cell number at previous time point – 1 (34).

### Function test and cytotoxicity assay

The T cells were subjected to co-culture with Thp-1, a monocyte cell line derived from the peripheral blood of a patient with acute monocytic leukemia (AML), at a ratio of 1:5 for 4 h to induce T-cell cytotoxicity [Thp-1 was labeled with carboxyfluorescein succinimidyl ester (CFSE)]. The cells were then harvested and stained with CD3 antibodies (BioLegend^®^). Following fixation with the Cyto-Fast^™^ Fix/Perm Buffer, TNF-α and IFN-γ were also stained with antibodies to evaluate the cytotoxicity of the T cells after cryopreservation in different media. The levels of TNF-α and IFN-γ in the SFSE^−^ and CD3^+^ populations were analyzed by flow cytometry. Furthermore, Thp-1 cell deaths were also quantified using 7AAD staining separately. The cell stemness and differentiation in the different cryopreservation media were assessed with CCR7 and CD45RA (both from BioLegend^®^), as described in the present research (35).

### Quantification and statistical analysis

Statistical analysis was performed using GraphPad Prism version 9.0 (GraphPad, San Diego CA, USA) or R software. For the comparison of two groups, Student’s *t*-test was used.

## Results

### The enhancement of T-cell viability preservation is notable when stored in C80EZ^®^ at a temperature of −80°C

**FIGURE 1:**
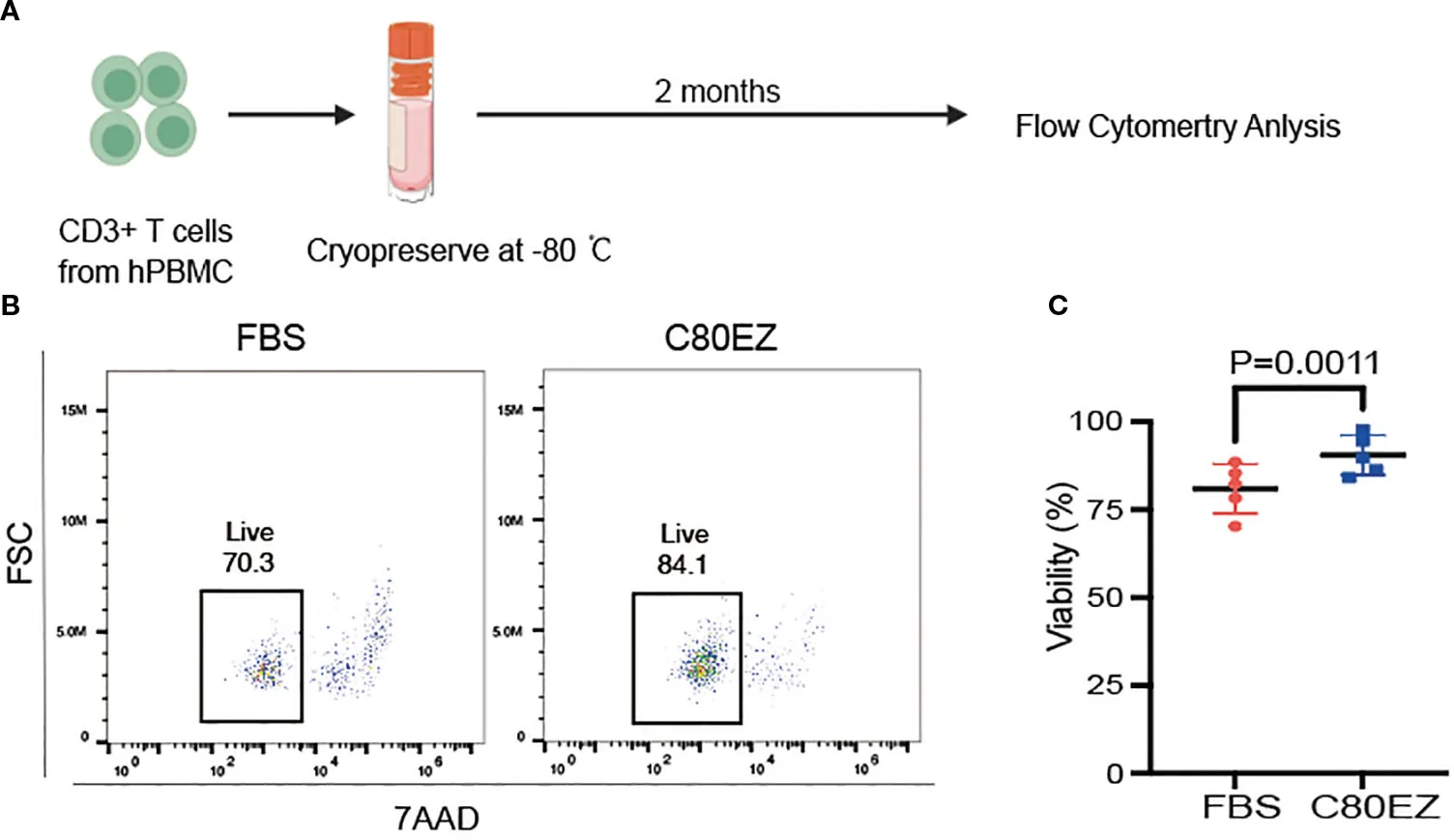
The cryopreservation medium C80EZ^®^ is more effective in maintaining the viability of T cells. **(A)** The process of T-cell cryopreservation (created in BioRender). **(B, C)** T-cell viability after cryo-storing at −80°C using different cryoprotectants (*left*: FBS; *right*: C80EZ^®^). Student’s *t*-test. *Error bars*, SEM.

To assess the viability of T cells in various cryopreservation media, we initiated the process by isolating T cells from human peripheral blood mononuclear cells (PBMCs). These cells were subsequently cryopreserved in a −80°C freezer using both the control medium (composed of 95% FBS and 5% DMSO) and C80EZ^®^ supplemented with 5% DMSO, with a duration of 2 months as the cryopreservation period (Figure 1A). After the designated freezing period in the experiment, all cells underwent recovery using the prescribed method. A consistent number of cells were then extracted for 7AAD staining, and subsequent analysis was carried out using a flow cytometer. The viability of the T cells in the C80EZ^®^ medium exhibited a significant increase (10% more live cells) compared with those in the FBS control group after 2 months of cryopreservation (Figures 1B, C). This observation supports the efficacy of C80EZ^®^ in maintaining the viability of T cells over an extended cryopreservation duration.

### The cryopreservation of T cells in C80EZ^®^ demonstrates superior proliferation compared to alternative methods

To explore the ability of the C80EZ^®^ medium to maintain T-cell proliferation, we extracted 1 × 10^5^ cells from each group and respectively cultured them in 96-well plates. The cell growth rate of T cells was then calculated at 48, 96, and 144 h post-seeding in the culture medium.

**FIGURE 2:**
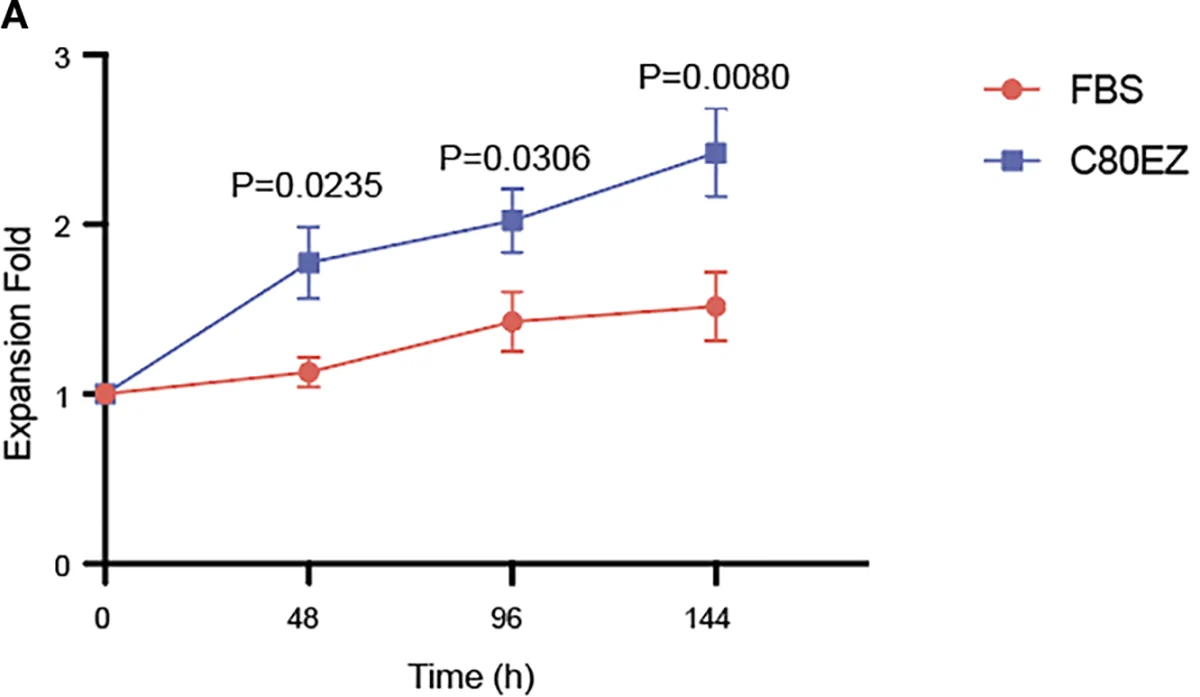
T cells preserved in C80EZ^®^ exhibit a higher growth rate after being thawed from −80°C. **(A)** T-cell proliferation assay data after thawing from −80°C using C80EZ^®^ and fetal bovine serum (FBS). *Error bars*, SEM. Expansion Fold, Harvest cell number/Cell number at previous time point – 1.

In alignment with the viability data, the growth trend of the cells in the C80EZ^®^ medium surpassed that in the FBS group (as depicted in Figure 2A). These findings indicate that the T cells preserved in the C80EZ^®^ medium maintained a heightened level of growth even after 2 months of cryopreservation. This underscores the effectiveness of C80EZ^®^ in not only sustaining viability but also promoting the proliferation capacity of the cryopreserved T cells.

### T cells preserved in C80EZ^®^ exhibit enhanced cytotoxicity and stemness

Our previous experiments confirmed that C80EZ^®^ not only maintains the cell vitality and proliferation ability but also crucially protects these functions through the incorporation of cryoprotectants (36).

**FIGURE 3:**
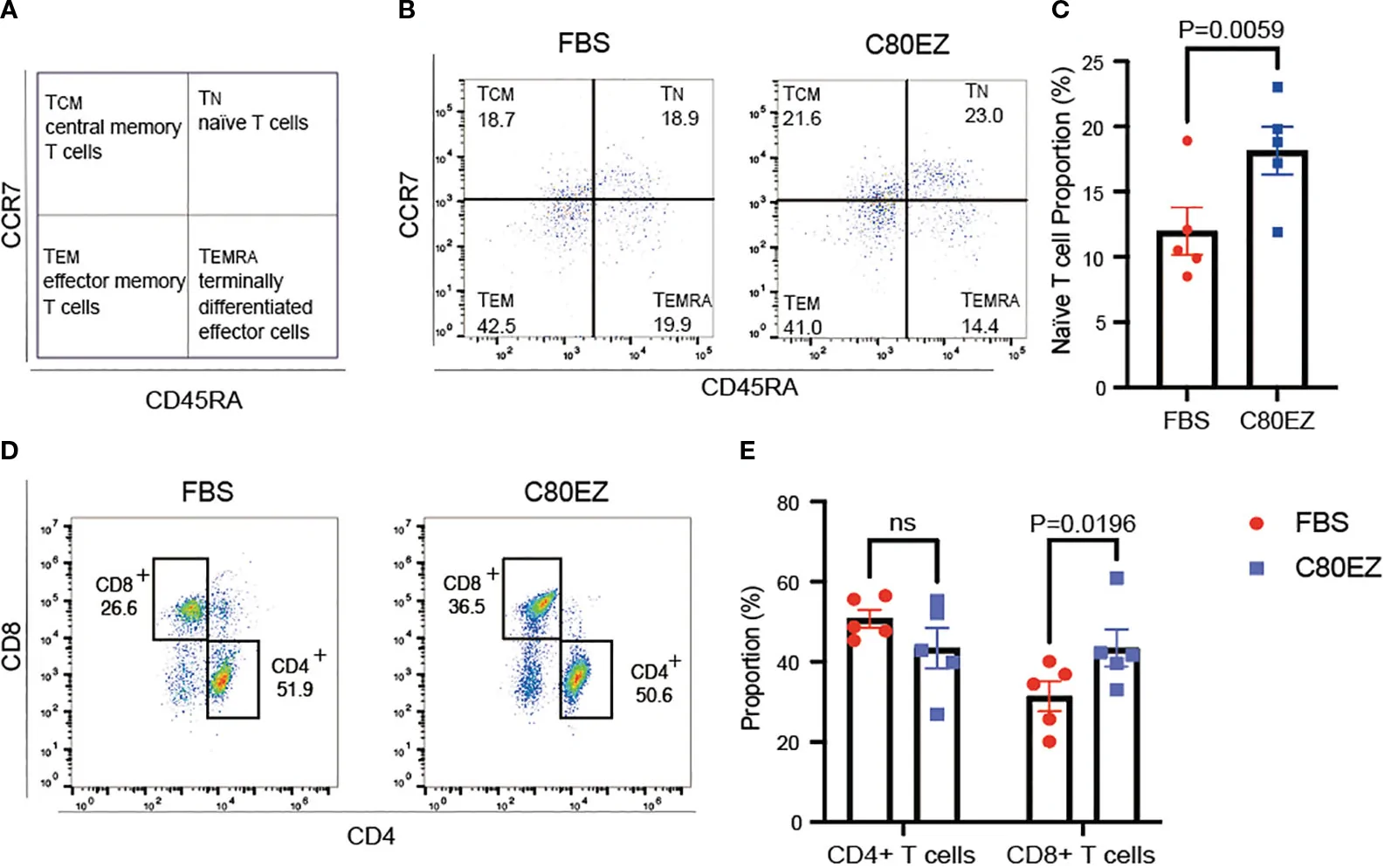
T cells preserved in C80EZ^®^ exhibit higher stemness and a higher proportion of the CD8^+^ subtype after storage at −80°C. (**A**) Gating strategy for identifying naive T cells (*TN*; CCR7^+^CD45RA^+^), central memory T cells (*TCM*; CCR7^+^CD45RA^−^), effector memory T cells (*TEM*; CCR7^−^ CD45RA^−^), and terminally differentiated effector cells (*TEMRA*; CCR7^−^ CD45RA^+^). (**B, C**) Representative flow cytometry data (**B**) and summary (**C**) showing higher T-cell stemness in the T cells cryopreserved using C80EZ^®^ at −80°C (*left*: FBS; right: C80EZ^®^). (**D, E**) Representative flow cytometry data (**D**) and summary (**E**) showing the different T-cell subtypes. Error bars, SEM. ns, No significance.

**FIGURE 4:**
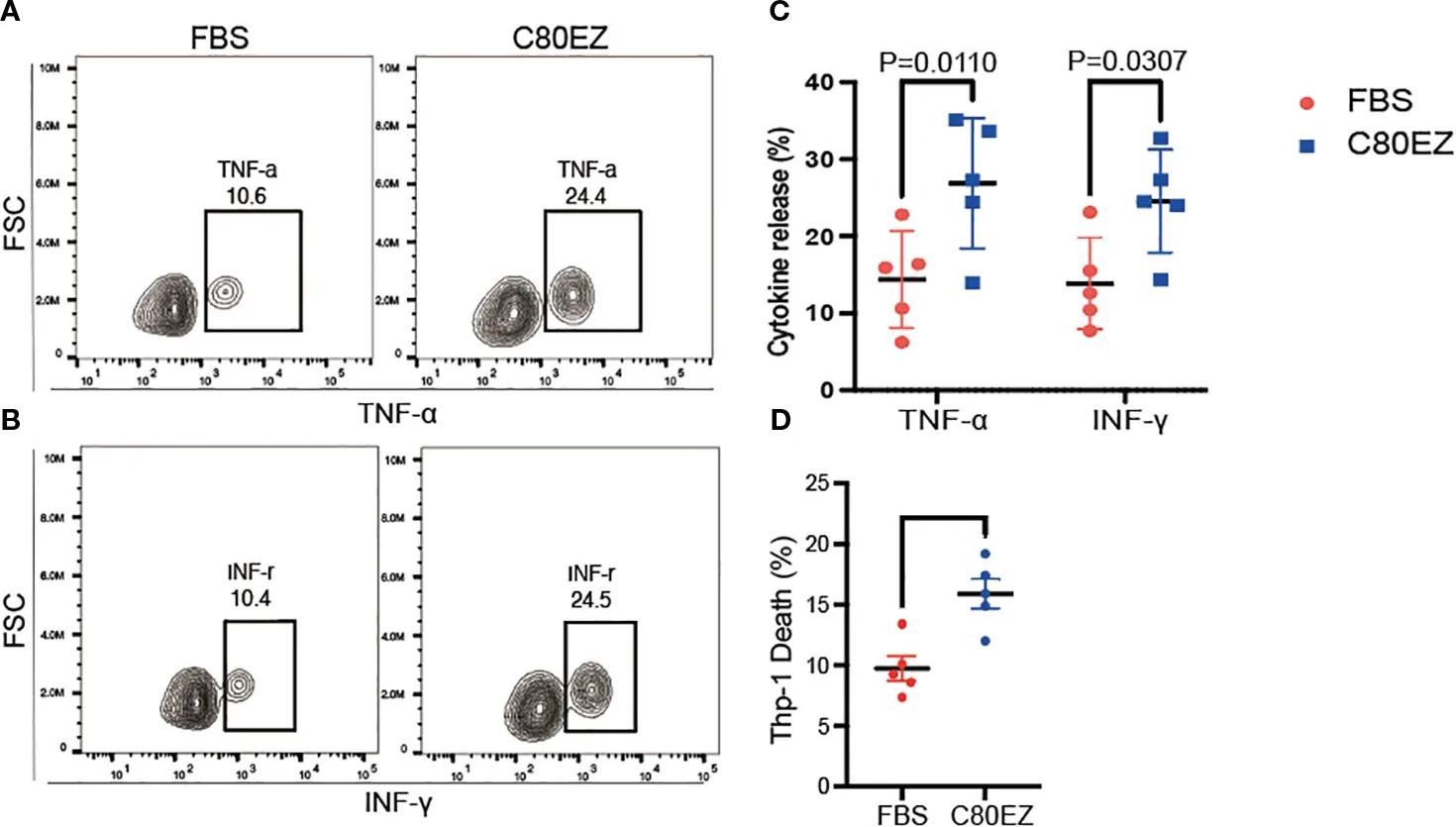
C80EZ^®^ at −80°C more effectively preserves the cytotoxicity of T cells. **(A–C)** Representative flow cytometry expression **(A, B)** and summary **(C)** of tumor necrosis factor alpha (TNF-α) and interferon gamma (INF-γ). **(D)** Death of the Thp-1 leukemia cell line co-cultured with the cryopreserved T cells from fetal bovine serum (FBS) or C80EZ^®^. *Error bars*, SEM.

In the evaluation of T cells, we conducted a series of tests for stemness, differentiation, and cytokine release to assess their functionality against tumor cells after cryopreservation at −80°C (Figures 3, 4). After 2 months of cryopreservation, the proportion of naive T cells in the C80EZ^®^ medium surpassed that in the other groups, as depicted in Figures 3A–C. Furthermore, the percentage of CD8^+^ T cells and the release of TNF-α and IFN-γ were notably higher in the C80EZ^®^ group compared with those in the other conditions (Figures 4A–C).

Moreover, C80EZ^®^ demonstrated the capability to maintain the proportion of CD8^+^ effector T cells within the total viable T-cell population after cryo-storage at −80°C, as illustrated in Figures 3D, E. To further investigate whether C80EZ^®^ preserves the cytotoxicity of T cells, we conducted a co-culture experiment with these T cells and Thp-1 AML cells. After incubating for 4 h at 37°C, we observed higher Thp-1 cell death in the C80EZ^®^ group (Figure 4D). These compelling findings substantiate that C80EZ^®^ stands as a superior cryopreservation medium for immune cells, not only ensuring cost-effective operations but also preserving their functional capacity against tumor cells.

## Discussion

Efficient long-term cryo-storage of regular mammalian cell types at −80°C relies on the efficacy in preventing ice recrystallization and its damaging effects. Our thermal and molecular dynamic studies have revealed that Ficoll molecules, a polysaccharide forming a unique, highly compact spherical shape, can completely prevent recrystallization and its damaging effects (25, 37, 38). In models using human embryonic and induced pluripotent stem cells, Ficoll-based cryopreservation at −80°C yielded post-thaw viability and proliferation efficiency comparable to that of the traditional use of FBS+DMSO and LN2 storage over 1 year. We also performed a viability comparison assay on the human T cells in C80EZ^®^ cryopreserved at −80°C and the T cells in FBS cryopreserved in LN2 for 2 months (Supplementary Figure S1A). The results showed that the T cells cryopreserved in these two conditions have comparable post-thaw viability. Our resulting commercial product, the C80EZ^®^ medium, also incorporates a low concentration of chondroitin sulfate as an anti-apoptotic agent to mitigate cell death from biophysical impacts such as intracellular water loss during freezing. C80EZ^®^ has been beneficial in various industrial applications, eliminating the need for serum in cell cryopreservation, reducing the DMSO concentration, and, most notably, removing the dependence on LN2 storage. Its effectiveness in preserving engineered insect cells over long-term storage at −80°C has also been confirmed (36).

This study aimed to highlight the superiority of C80EZ^®^ by comparing it with high-concentration FBS-based methods. Although commercial media such as CryoStor^®^ CS10 are used clinically, especially for T-cell cryopreservation, their post-thaw viability and functionality fall interior in comparison to the use of high FBS concentrations (e.g., 95%); moreover, they are not recommended for long-term −80°C storage (39). The primary advantage of CS10 is the exclusion of animal serum, addressing regulatory concerns. Hence, our study will focus on comparing C80EZ^®^ with an FBS+DMSO control.

There have also been several studies into novel cryoprotectants of T cells that have shown exciting results in terms of the high viability and the cytotoxicity of post-thaw T cells (40, 41), but they have all focused on reducing the cytotoxicity of DMSO or replacing FBS in the cryopreservation medium. On the other hand, our study was dedicated to replacing LN2 with higher temperatures, which can be achieved with general equipment.

In our control group using an FBS-based medium, similar to previous studies on the storage of T cells in LN2, the cell proliferation and toxicity were affected (42, 43). However, the effects observed in our control group were more pronounced than those seen with LN2 storage. This difference could be attributed to cellular damage associated with the recrystallization process in −80° C freezers, a phenomenon also noted in other cell types stored at −80°C (28). Conversely, the results from our study revealed similar viability of the T cells stored in C80EZ^®^ at −80°C to those cryopreserved in LN2, indicating that C80EZ^®^ significantly improved the protection ability during the cryopreservation, similar to its effects on engineered insect cells or stem cells (25, 36).

The examination of T-cell functionality, encompassing cytotoxicity, stemness, differentiation, and TNF-α and IFN-γ release after cryopreservation at −80°C, solidifies the superiority of C80EZ^®^. The proportion of naive T cells, the percentage of CD8^+^ T cells, and the release of TNF-α and IFN-γ were notably higher in the C80EZ^®^ group compared with that in other conditions. Moreover, C80EZ^®^ demonstrated the capability to maintain the proportion of CD8^+^ effector T cells within the total viable T-cell population after cryo-storage at −80°C. These findings collectively substantiate the adoption of C80EZ^®^ as a potentially superior choice for biobanking immune cells. Ongoing studies are in progress to confirm the efficacy of longer-term storage.

However, although we have performed a series of studies on the biological parameters, the current study is limited to the 2-month storage period. Based on our research with stem cells, the reduction in post-thaw viability would likely be significantly more pronounced if a longer storage period were studied. No viability would logically result in null proliferation and functionality. Long-term storage will be evaluated to substantiate our prediction, as a future direction. Moreover, through our proliferation results, despite the use of the C80EZ^®^ medium, post-thaw proliferation remains suboptimal, indicating that this issue may not be resolved solely by enhancing the cryopreservation medium. Instead, it could potentially be addressed by refining the post-thaw culturing protocols, such as the inclusion of Rho-associated protein kinase (ROCK) inhibitors (44) or other agents or the improvement of the culture conditions.

In conclusion, our study provides valuable insights into overcoming challenges in T-cell cryopreservation, introducing C80EZ^®^ as an innovative solution to enhance T-cell viability, proliferation, and functionality. This holds significant promise for advancing the efficacy and accessibility of T-cell-based immunotherapies, as the technology addresses prevalent practical and technical challenges in quality control and supply chain management associated with the therapeutic process.

## Supplementary Material

Supplementary

The Supplementary Material for this article can be found online at: https://www.frontiersin.org/articles/10.3389/frhem.2024.1346627/full#supplementary-material

## Data Availability

The raw data supporting the conclusions of this article will be made available by the authors, without undue reservation.
