# Supplementary material for: Novel cryopreservation medium for enhanced stability of T cells at −80°C
Source: Front Hematol. Author manuscript; Available in PMC 2026 Sep 15. (PMC13573834; doi:10.3389/frhem.2024.1346627)
Supplement: Supplementary file 1 [file NIHMS2206131-supplement-Supplementary.docx]

Supplemental Figure 1

A

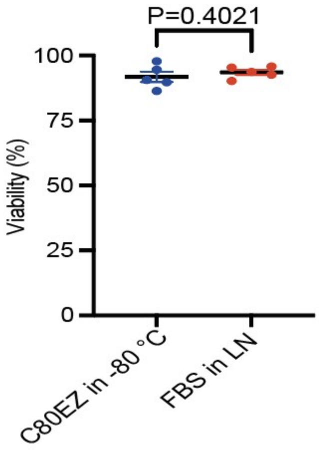

Supplemental Figure 1 A. The viability comparison of T cells in C80EZ cryopreserved at -80 °C and T cells in FBS cryopreserved in LN. Error bar. sem.
